# RNA interference machinery-mediated gene regulation in mouse adult neural stem cells

**DOI:** 10.1186/s12868-015-0198-7

**Published:** 2015-09-19

**Authors:** Filippo M. Cernilogar, Rossella Di Giaimo, Frederick Rehfeld, Silvia Cappello, D. Chichung Lie

**Affiliations:** Research Group Adult Neurogenesis and Neural Stem Cells, Institute of Developmental Genetics, Helmholtz Center Munich, German Research Center for Environmental Health, Munich-Neuherberg, Germany; Biomedical Center, Ludwig Maximilian University, Großhaderner Strasse 9, 82152 Planegg-Martinsried, Germany; Institute for Stem Cell Research, Helmholtz Center Munich, German Research Center for Environmental Health, Munich-Neuherberg, Germany; Department of Biology, University of Naples Federico II, Naples, Italy; Institute of Cell Biology and Neurobiology, Charité University, Berlin, Germany; Institute of Biochemistry, Emil Fischer Center, Friedrich-Alexander-Universität Erlangen-Nürnberg, Erlangen, Germany; Developmental Neurobiology, Max Planck Institute of Psychiatry, Munich, Germany

**Keywords:** RNAi, Dicer, Argonaute, Doublecortin, miRNA-128, Adult neurogenesis

## Abstract

**Background:**

Neurogenesis in the brain of adult mammals occurs throughout life in two locations: the subventricular zone of the lateral ventricle and the subgranular zone of the dentate gyrus in the hippocampus. RNA interference mechanisms have emerged as critical regulators of neuronal differentiation. However, to date, little is known about its function in adult neurogenesis.

**Results:**

Here we show that the RNA interference machinery regulates Doublecortin levels and is associated with chromatin in differentiating adult neural progenitors. Deletion of Dicer causes abnormal higher levels of Doublecortin. The microRNA pathway plays an important role in Doublecortin regulation. In particular miRNA-128 overexpression can reduce Doublecortin levels in differentiating adult neural progenitors.

**Conclusions:**

We conclude that the RNA interference components play an important role, even through chromatin association, in regulating neuron-specific gene expression programs.

**Electronic supplementary material:**

The online version of this article (doi:10.1186/s12868-015-0198-7) contains supplementary material, which is available to authorized users.

## Background

The precise regulation of proliferation, survival, migration and differentiation of neural stem cells (NSCs) and neural progenitors is crucial for proper formation of the mammalian brain during embryonic, postnatal and adult stages [1–5]. In the adult mouse brain, NSCs persist throughout life in the subgranular zone of the hippocampal dentate gyrus (DG) and the subventricular zone (SVZ) of the lateral ventricle. In these regions, NSCs generate mature neurons through a complex sequence of developmental steps including the division of a precursor cell and a multi-step process (proliferation, differentiation, migration, targeting, and synaptic integration) that ends with the formation of a postmitotic functionally integrated new neuron [6]. These developmental stages can be distinguished on the basis of the expression of stage-specific marker proteins including the microtubule binding protein Doublecortin (Dcx). In the adult mouse brain, Dcx is almost exclusively expressed by immature newborn neurons in the DG and the SVZ/Olfactory Bulb-system and is commonly used to distinguish immature neurons from non-neuronally committed precursors and to estimate neurogenic activity [7–10]. Perturbation of adult neurogenesis is associated with learning memory deficits, dysregulation of mood, and has been implied in the pathogenesis of neuropsychiatric disorders [11, 12]. Hence, revealing the regulation of genetic programs crucial for NSCs expansion and lineage commitment in the adult brain can provide the molecular basis for developing effective methods for NSC-based and adult neurogenesis-targeted therapies.

RNA interference (RNAi) mechanisms are a well-conserved phenomenon in eukaryotes and act at multiple levels to regulate gene expression [13–16]. A large number of small RNA classes have been identified, including microRNAs (miRNAs), small interfering RNAs (siRNAs) and Piwi-interacting RNAs (piRNAs). These classes differ in their biogenesis, their modes of target regulation and in the biological pathways they regulate [17–19]. In mammals small RNA silencing pathways depend on two groups of genes encoding the key proteins of the Dicer and Argonaute families [18].

The RNase III enzyme Dicer is required for the processing of short (21–22 nucleotides) micro-RNAs (miRNAs) and small interfering RNAs (siRNAs) from double stranded RNA precursors. Dicer-generated RNAs trigger the degradation of complementary mRNAs or prevent their translation [13–15]. In addition accumulating evidence indicates that RNAi components and small RNAs participate in nuclear processes such as transposon regulation, heterochromatin formation, genome stability and transcription [20–23]. Defining the role of Dicer generated small RNAs in mammalian development is complicated by embryonic lethality of constitutive Dicer knockouts in mice [24, 25]. Mouse embryonic stem cells in the absence of Dicer fail to differentiate in vitro and do not contribute to mouse development in vivo [26]. This highlights the importance of siRNAs and miRNAs in the regulation of gene expression during cell differentiation.

Here we show that the RNAi machinery regulates Dcx levels and is associated with chromatin in differentiating adult neural progenitors. Deletion of Dicer causes abnormal higher levels of Dcx expression and miRNA-128 over-expression can down-regulate Dcx levels in differentiating adult neural progenitors. In addition a chromatin-binding assay reveals that some of Dicer and Argonaute-2 associate to chromatin in differentiating adult neural progenitors. We conclude that the Dicer/Argonaute-2 RNAi pathway plays an important role, potentially even through chromatin association, in regulating gene expression programs in the neural stem cell lineage.

## Results

### Adult neural stem cells lacking Dicer have higher level of Doublecortin

**Fig. 1:**
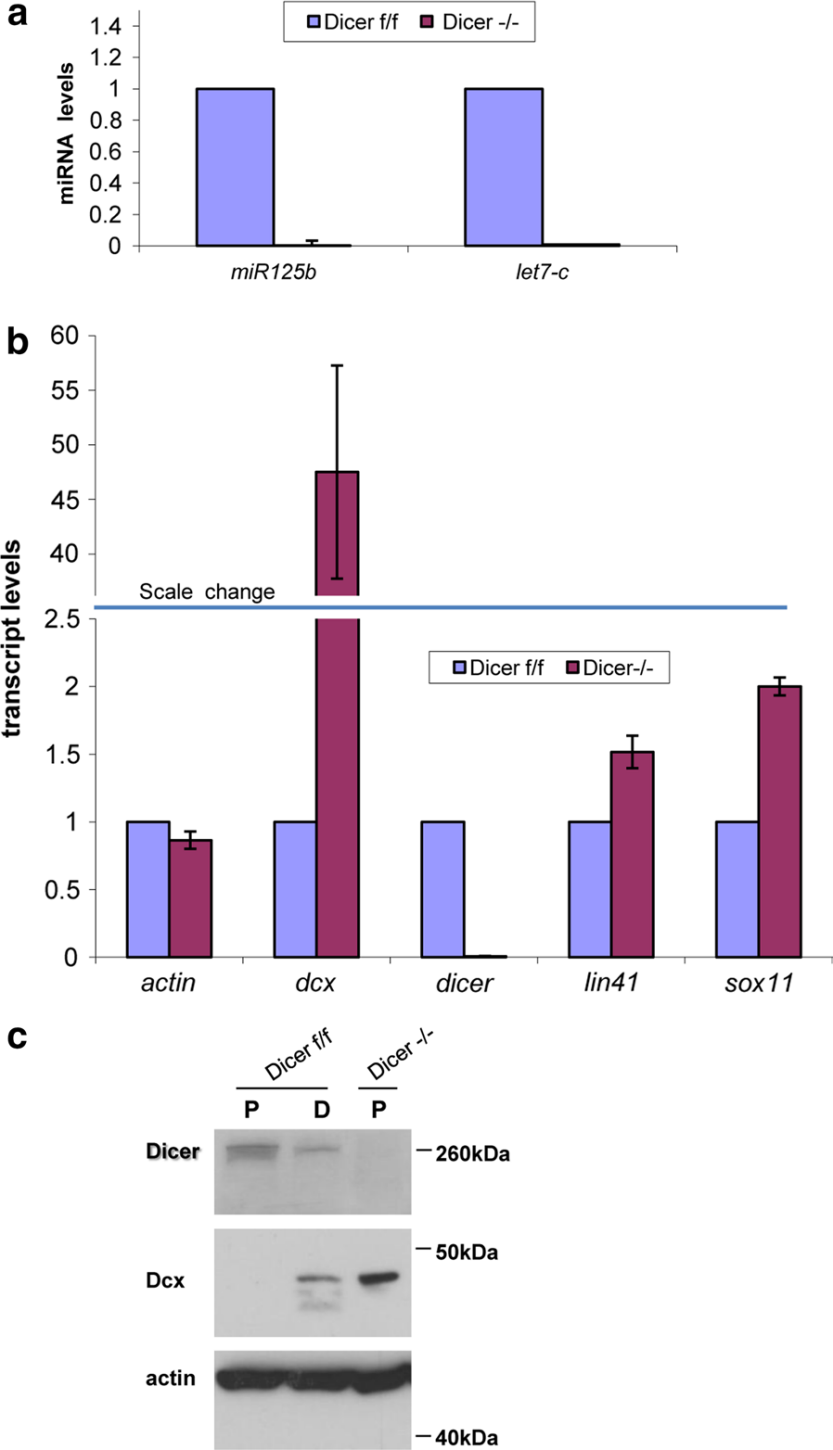
Dcx is up-regulated upon Dicer deletion in adult neural stem cells. **a**–**c** The samples analyzed were adult neural progenitor cells (NPCs) *Dicer flox*/*flox* (*f*/*f*) *or Dicer* −/−. *Dicer* deletion was obtained by transducing *Dicer f*/*f* cells with HTN-Cre protein. **a** Taqman quantitative RT-PCR of *miR125b and let7*-*c* mature miRNA transcripts. Transcript levels were normalized against the housekeeping gene *snoRNA55* and calculated respect to *Dicer f*/*f* (control) that is set to 1. n = 3, *bars* represent the mean ± standard error of the mean. **b** Quantitative RT-PCR of the indicated transcripts. Transcript levels were normalized against the housekeeping gene *Srp14* and calculated respect to *Dicer f*/*f* (control) that is set to 1. The *blue horizontal bar* indicates the scale change in the *y-axis*. n = 3, *bars* represent the mean ± standard error of the mean. **c** Western blot showing a representative *picture* of the Dicer, Dcx and Actin (loading control) protein levels in NPCs (*P*) or differentiating NPCs (*D*; 4 days after growth factor withdrawal) of the indicated genotype. Most of the *Dicer* −/− cells die upon induction of differentiation and therefore are not included in the analysis. Three independent biological samples have been analyzed. Shown are representative pictures

To study RNAi machinery function in mouse adult neural stem cells we isolated neural progenitor cells (NPCs) from the SVZ of 8 weeks old *Dicer cKO* (*Dicer*^*flox/flox*^) mice [27]. The deletion of the LoxP-flanked exons 20–21 in the *Dicer* locus was induced by transducing NPCs with the HTN-Cre protein, a recombinant fusion protein able to cross the cell and nuclear membrane [28]. Consistent with the almost complete deletion of the *Dicer* locus (Additional file 1: Fig. S1A) the HTN-Cre treated cells showed a marked decrease in the levels of mature miRNAs (Fig. 1a), of *Dicer* transcript (Fig. 1b), and of Dicer protein (Fig. 1c) indicating that activity of the RNAi machinery was severely impaired. Along with the *Dicer* transcript we analyzed the levels of additional transcripts (Fig. 1b), namely *Actin* as a housekeeping gene, *Lin41* as miRNA-dependent gene [29], *Dcx* and *Sox11* as possible miRNA targets based on the miRNA target prediction algorithm TargetScan [30]. The *Actin* transcript levels were almost unchanged; *Lin41* and *Sox11* transcripts were slightly up-regulated, but strikingly *Dcx* showed a 47.5 (±9.7 SEM) fold increase in *Dicer* null cells compared to control cells. Then we asked if the increase of the *Dcx* transcript in *Dicer* null cells resulted also in an increase of the Dcx protein level. In control NPCs, the Dcx protein was detectable only in differentiating cells (Fig. 1c) but, consistent with the qPCR data, the *Dicer* −/− NPCs had already detectable Dcx protein. Most of the *Dicer* −/− NPCs die upon induction of differentiation and therefore this condition was not included in the further analysis.

Overall our results show that in NPCs the ablation of *Dicer* and the consequent impairment of the RNAi machinery result in the up-regulation of the Dcx transcript and protein.

### Doublecortin is upregulated upon Dicer deletion in hippocampal newborn neurons

**Fig. 2:**
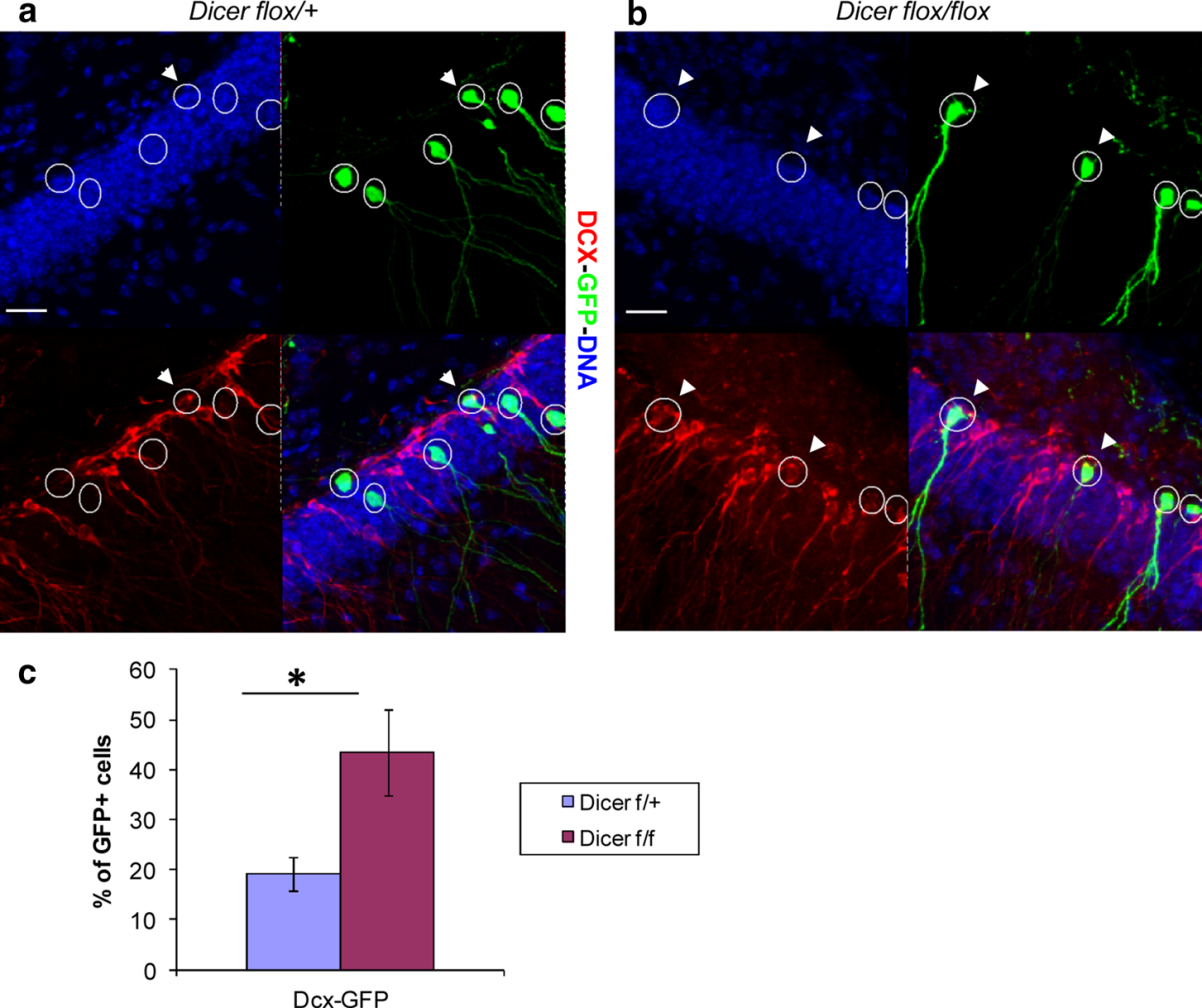
Dcx is up-regulated upon Dicer deletion in hippocampal new born neurons. **a**, **b** Representative *pictures* of the immunostaining for Dcx and GFP on hippocampal regions of *Dicer cKO* mice transduced with Cre-recombinase. **a** *Dicer flox*/+ mice (control) 28 dpi. **b** *Dicer flox*/*flox* mice (experiment) 28 dpi. Transduced cells are GFP positive (*green*) and identified by *white circles*. *Arrowheads* indicate Dcx-expressing (*red*) among the Cre-transduced (*green*) cells. Single channel and overlay *pictures* are shown. *Scale bar* 25 µm. **c** Quantification of Dcx/GFP double positive cells among the virus-transduced cells. *Bars* show the average ± the standard error of the mean (n = 4). At least 100 virus-transduced cells per group of four animals were analyzed. Two-tailed t test was applied for statistical analysis. *Asterisks* indicate statistically relevant differences; p < 0.05

In order to understand if the RNAi machinery impairment can deregulate Dcx expression also in the in vivo context we induced *Dicer* deletion in the adult hippocampal neurogenic lineage by injecting adult (8 weeks old) *Dicer cKO* (*Dicer*^*flox*/*flox*^) mice with a recombinant mouse moloney leukemia-retrovirus bicistronically encoding for GFP and Cre-recombinase into the septal hippocampal dentate gyrus. Heterozygous *Dicer cKO* (*Dicer*^*flox*/+^) mice injected with the same retroviral preparation served as controls. Animals were sacrificed at 28 days post injection (dpi). Brain sections were immunostained for the immature neuronal marker Dcx and for GFP, to identify virus-transduced cells (Fig. 2a, b). Consistent with previous studies most of virus-transduced cells in heterozygous *Dicer cKO* readily down regulated Dcx at 28 dpi [31, 32]. Interestingly, we found a significant increase (Fig. 2c) of Dcx/GFP double-positive cells in the *Dicer cKO.* We also checked another immature neuronal marker, Sox11, but did not observe significant differences of Sox11/GFP double positive cells in the *Dicer cKO* respect to the control (control: 49.6 % ± 11.3 SEM; Dicer cKO: 63.9 % ± 9.7 SEM; n = 4, two-tailed *t* test p = 0.4). The lack of significant change in Sox11 expression is in line with our in vitro analysis (Fig. 1b) where *Dicer* −/− NPCs show minor increase in Sox11 expression. Additionally we observed a consistent reduction (about 40 %) of virally transduced GFP positve cells in the *Dicer cKO* (not shown). Although in the present experimental set up we cannot fully exclude differences in the virus transduction efficiency between different experimental groups this would be suggestive of reduced viability of Dicer deficient neurons. Taken together these data indicate that in newborn neurons Dcx levels are sensitive to the levels of Dicer and thus to the functionality of the RNAi machinery.

### Overexpression of miRNA-128 reduces Doublecortin levels in differentiating adult neural stem cells

**Fig. 3:**
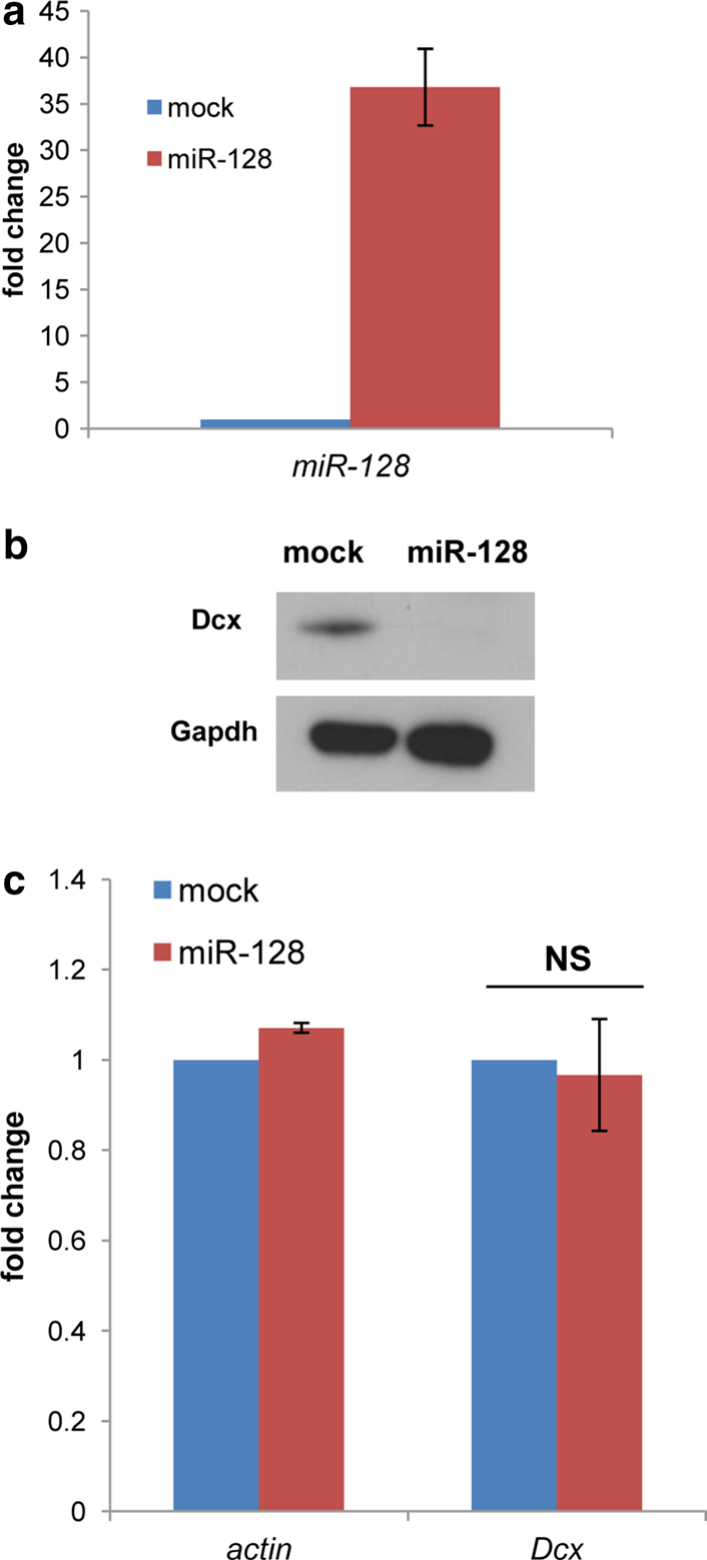
Overexpression of miRNA128 reduces Dcx protein levels in N2A cells. **a**–**c** The samples analyzed were mock-treated Neuro2A cells (control transfection without miRNA-128-RFP expressing plasmid) or Neuro2A cells treated with miRNA-128-RFP expressing plasmid. **a** Taqman quantitative RT-PCR of the mature miRNA-128 transcripts normalized against the housekeeping small non-coding RNA *snoRNA55*. Transcript levels are indicated as fold change respect to the control (mock) that is set to 1. n = 3, *bars* represent the mean ± standard error of the mean. **b** Western blot showing the Dcx and Gapdh (loading control) protein levels in mock and miRNA-128 over-expressing cells. **c** Quantitative RT-PCR of the indicated transcripts. Transcript levels are indicated as fold change respect to the control (mock) that is set to 1. Transcripts were normalized against the housekeeping gene *Srp14*. n = 4, *bars* represent the mean ± standard error of the mean. Two-tailed t test was applied for statistical analysis. *NS* not significant; p = 0.4

**Fig. 4:**
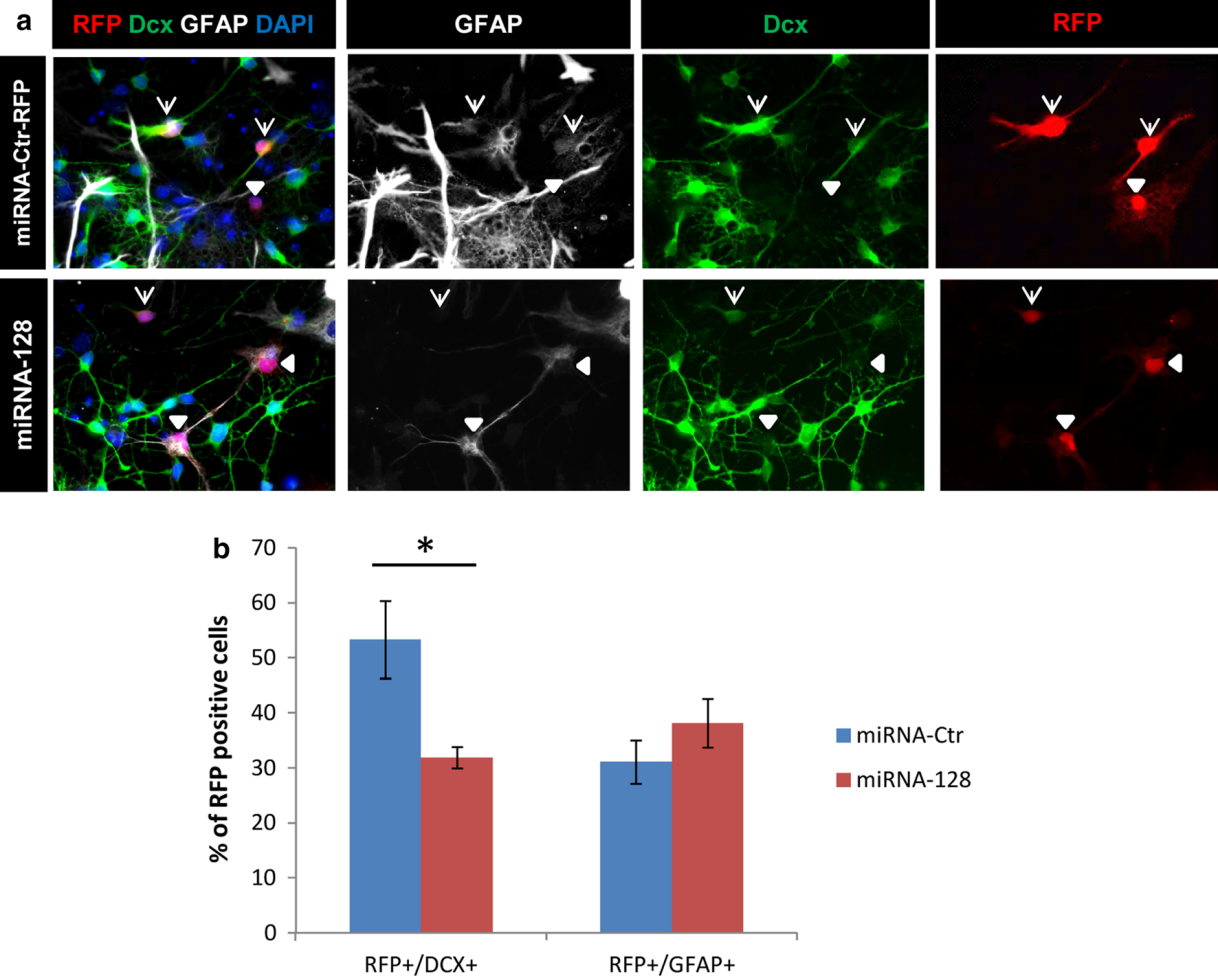
Overexpression of miRNA128 reduces Dcx protein levels in “in vitro” differentiating adult neural stem cells. **a**, **b** Adult neural progenitor cells were transfected with miRNA-Ctr-RFP (control) or mirRNA-128-RFP (experiment) expressing plasmids and induced to differentiate by growth factor withdrawal. Analysis was done 6 days later. **a** Representative *pictures* of the immunostaining of Dcx, GFAP and RFP on control and experiment cells. DAPI visualizes the DNA. Transfected cells are RFP positive. *Arrows* indicate Dcx/RFP positive cells; *arrowheads* indicate GFAP/RFP positive cells. **b** Quantification of Dcx and GFAP among the transfected cells. *Bars* show the average ± the standard error of the mean (n = 3). The percentage of marker-positive cells among the total RFP-positive population was calculated on three biological replicates. At least 100 cells per experimental group were analyzed. Two-tailed t test was applied for statistical analysis. *Asterisks* indicate statistically relevant differences; p < 0.05

Our in vitro and in vivo (Figs. 1, 2) experiments show that the impairment of the RNAi machinery through the deletion of *Dicer* in NPCs is paralleled by abnormally elevated levels of Dcx. The Dcx transcript has been shown to be regulated by the RNAi machinery through miRNA-dependent pathways that are severely reduced upon *Dicer* deletion (Fig. 1a). Specifically, miR-134 [33] was shown to regulate Dcx in mouse embryonic brain tissues, while miR-128 [34] was found to modulate Dcx levels in a human neuroblastoma cell line. Of these, we found only miR-128 to be expressed in adult mouse NPCs (Additional file 2: Fig. S2) in agreement with a previous report [35] showing the presence of miR-128 but not miR-134 in the neurogenic areas of the mouse adult brain. We next asked whether miR-128 could target and regulate Dcx levels in adult NPCs. To this purpose we created a miR128-RFP expression vector (see “Methods”). To check its functionality we transfected this vector into Neuro2A cells, which resulted in a marked increase in miR-128 levels (Fig. 3a) along with decreased levels of Dcx protein but not the transcript (Fig. 3b, c; Additional file 3: Fig. S3). This indicates that the vector is able to increase the miR-128 levels leading to Dcx down-regulation by translational repression as it has been described for many miRNAs [36]. We then transfected the miR-128-RFP or miR-Ctr-RFP (negative control containing a scrambled miRNA sequence) in adult NPCs (derived from the SVZ of 8-weeks old mice) and let them differentiate for 6 days. After fixation the cells were immunostained for Dcx, GFAP (a marker for astroglial cells) and RFP marking the transfected cells (Fig. 4a). Interestingly, we found a significant decrease in the fraction of Dcx positive cells among the transfected cells when miR-128 is overexpressed (Fig. 4b). Notably, the fraction of GFAP positive cells among the transfected ones was not significantly affected. Our results show that miR-128 overexpression reduces the levels of Dcx in differentiating NPCs indicating that miR-128 can target and potentially take part in the regulation of Dcx levels in adult neurogenesis.

### RNAi components are associated with chromatin in differentiating adult neural stem cells

**Fig. 5:**
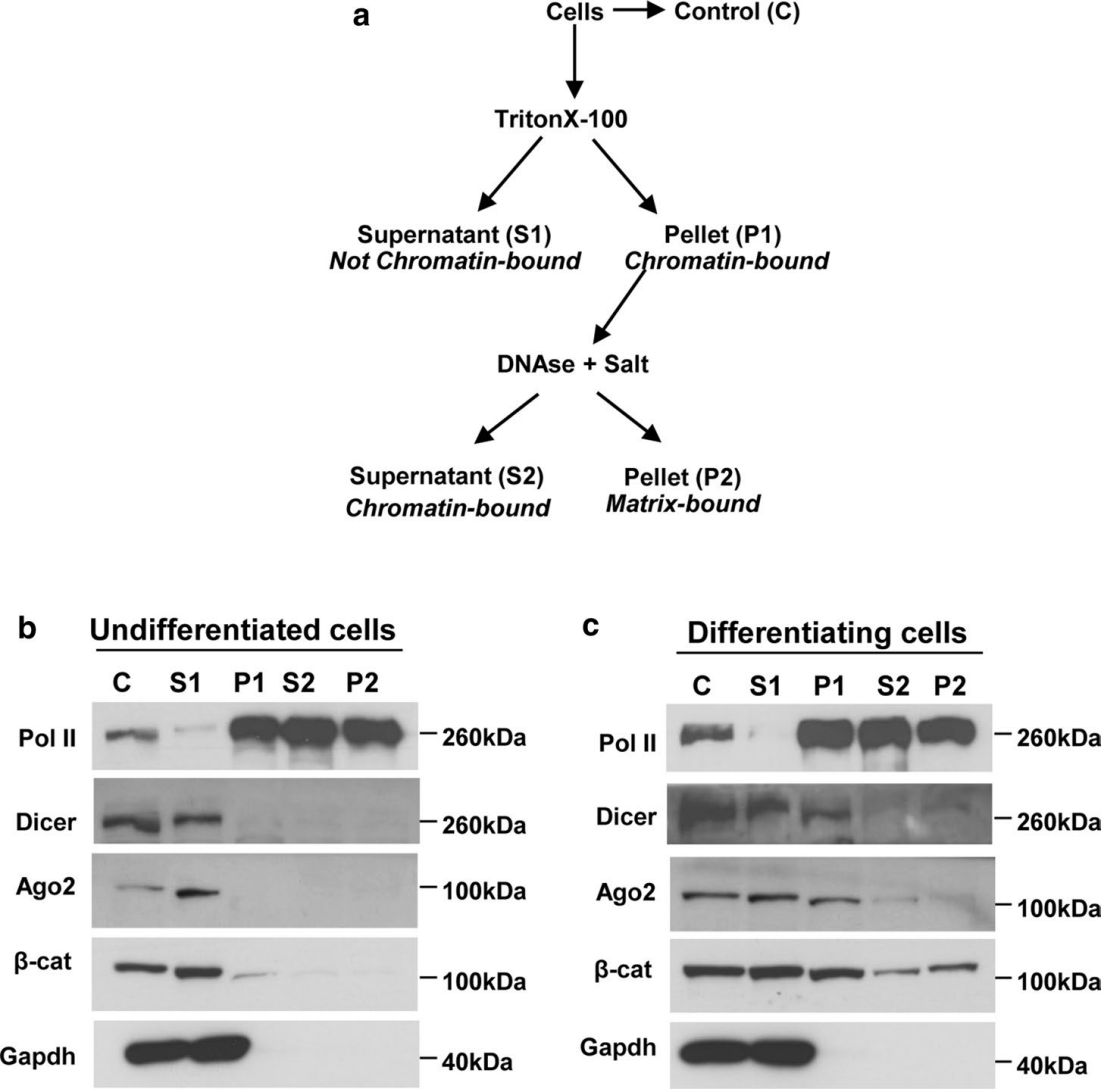
RNAi components are associated with chromatin in differentiating adult neural stem cells. **a** Scheme of the procedure followed to fractionate neural progenitor cells. Insoluble fractions (pellets) were dissolved in RIPA buffer (see “Methods”). Chromatin associated proteins should be found in fractions P1 and S2. **b**, **c** Equivalent amounts of the resulting protein samples from undifferentiated or differentiating (3 days) adult neural progenitor cells were analyzed by western blot for the presence of the indicated proteins. Gapdh (glyceraldehyde 3-phosphate dehydrogenase) serves as a chromatin unbound marker; Pol II (RNA Polymerase II) is a chromatin bound marker. *β-cat* β-catenin, *Ago2* Argonaute-2. Three independent biological samples have been analyzed. Shown are representative *pictures*

The RNAi machinery regulates gene expression mainly in the cytoplasm but has been reported to act also in the nucleus [20, 22, 23, 37, 38]. In order to assess if the main RNAi components could operate in the nucleus of NPCs we used a chromatin binding assay [23, 39] on undifferentiated and differentiating NPCs. This assay has been already used by us and others to predict the chromatin association of RNAi components [22, 23]. Chromatin associated proteins will be found in TritonX-100 resistant (P1) and, subsequently, in DNase and high salt (S2) extracted fractions (Fig. 5a). Indeed, a substantial portion of RNA Polymerase II (Pol II) is detected in these fractions (Fig. 5b). In contrast, most of Dicer and Argonaute-2 (Ago2) are found in the TritonX-100 soluble fraction (S1), along with β-catenin and glyceraldehyde 3-phosphate dehydrogenase (Gapdh), a marker for proteins not associated with chromatin (Fig. 5b). In differentiating NPCs (Fig. 5c) the markers Pol II and Gapdh are found in the same fraction as in undifferentiated cells. In contrast to undifferentiated NPCs, β-catenin was detected also in the chromatin fractions (P1, S2) consistent with the activation of the Wnt pathway during NPCs differentiation [40–42]. Interestingly, also Dicer and Ago2 display a differential fractionation profile depending on the differentiation status of the NPCs; thus Dicer and Ago2 were also found in the chromatin fraction P1 in differentiating NPCs. In contrast to Pol II and β-catenin, Dicer and Ago2 are found almost exclusively in the P1 but barely in the S2 chromatin fraction indicating that TritonX-100 is sufficient to fully release these proteins most likely due to a looser chromatin association of Dicer and Ago2 compared to Pol II and β-catenin.

Thus, the Dicer/Ago2 complex displays differential association with chromatin of adult NPCs depending on their differentiation status, raising the possibility that nuclear RNAi may contribute to the control of gene expression during specific developmental stages of adult neurogenesis.

## Discussion

The RNAi machinery has emerged as an important regulator of neuronal differentiation but to date its contribution in adult neurogenesis is still not fully understood. We further probed the function of the RNAi machinery by loss-of-function, gain-of-function and biochemical approaches.

We show that in adult NPCs the ablation of Dicer results in a massive up-regulation of the Dcx transcript and protein (Figs. 1, 2). This suggests that Dcx levels are heavily influenced directly or indirectly by RNAi activity. In favor of a direct regulation, *Dcx* mRNA has been shown to be targeted by different miRNAs [33, 34], which—in absence of Dicer—would not be matured and consequently would not be able to target *Dcx* mRNA for degradation or translational inhibition. In line with this view we show that overexpression of the miR-128 in differentiating adult NPCs causes the reduction of the Dcx levels (Fig. 4). However miR-128 and Dcx are not mutually exclusive in our in vitro cultured NPCs. Indeed miR-128 maintains similar level of expression also in differentiating NPCs (Additional file 2: Fig. S2) when Dcx is physiologically expressed (Fig. 1c). Like many miRNAs, miR-128 has been show to have relatively modest effects on its targets [43]. Thus, in differentiating NPCs, miR-128 is likely responsible to fine-tune rather than switching off Dcx, by repressing but without eliminating it. Notably the miRNA algorithm TargetScan (http://www.targetscan.org) identifies, in addition to miR-128, let-7 and miR-29 as potential regulators of Dcx. Both these miRNA are, similarly to miR-128, expressed in the neurogenic areas of the adult mouse brain [35] making them possible additional regulators of Dcx expression.

Argonaute directly binds the mature miRNA processed by Dicer and seeks target mRNAs that have complementarity to the miRNA. If complementarity exists in the central region of the miRNA (nucleotides 9–11) then the mRNA target can be cleaved via the endonuclease activity of Ago2. Differently Argonaute is recruited to a complex containing GW182 within cytoplasmic P bodies where translational repression occurs [36]. Notably miR128 lacks this complementarity (nucleotides 9–11) in *Dcx* target sites predicted by the miRNA algorithms TargetScan and microRNA.org [44] (http://www.microrna.org). In agreement with this prediction we found only the Dcx protein but not the transcript being down regulated upon miR128 overexpression (Fig. 3). Therefore the massive up-regulation of the *Dcx* transcript observed in NPCs upon *Dicer* deletion (Fig. 1) is hardly explained only by a decrease of miRNAs targeting *Dcx* but it likely involves additional layers of RNAi-dependent regulation.

miRNAs have been implied in the modulation of differentiation and proliferation of adult NPCs [45–50]. We kept adult *Dicer*-null NPCs in culture for weeks without visible defects but we observed a massive cell death upon stimulation of differentiation by growth factor withdrawal (data not shown), arguing that in particular differentiating NPCs are highly dependent on Dicer. In line with a particular importance of Dicer for differentiating cells, previous studies [51, 52] showed that Dicer is strictly required for embryonic NPCs differentiation. The ability of *Dicer*-null NPCs to generate both neurons and glia was, however, restored by the reintroduction of Dicer, demonstrating that these cells were not irreversibly transformed despite the absence of Dicer and microRNAs [51]. Similarly, reintroduction of Dicer was able to rescue the differentiation impairment of mouse embryonic stem cells *Dicer* −/− [53]. The dependency of NPCs differentiation on Dicer may not only be related to Dicer’s crucial function in miRNA biogenesis. RNA interference components including Dicer have been implicated in nuclear related functions such as transposon regulation, heterochromatin formation, genome stability and transcription [20–23]. Notably, by using a chromatin binding assay we observed that some of the key RNAi components, Dicer and Argonaute-2, associate to chromatin in differentiating adult NPCs (Fig. 5). Although a ChIP-seq experiment (chromatin immunoprecipitation followed by next generation sequencing) will be required to reveal the Dicer and Ago-2 binding sites on chromatin, this already suggests that the critical role of RNAi in adult NPCs differentiation might be also linked to its chromatin-association.

Interestingly, transposable elements are expressed and active in the brain. LINE-1 elements, for example, are actively mobilized in normal mammalian brains during neurogenesis [54–56], leading to genetic heterogeneity, which might be of functional relevance. However, because transposable elements are capable of jumping into new positions in the genome they are potentially detrimental. Transposable element activation has been correlated with several neurodegenerative diseases [57–64]. Interestingly it has been recently shown that mutations in the RNAi key component Argonaute-2 resulted in increased transposons expression in the Drosophila brain leading to detrimental effects such as accelerate memory impairment and shortened life span [65]. It is tempting to speculate that RNAi components might have a similar function in the mammalian brain by acting as a barrier against excessive transposable element activity during neurogenesis.

## Conclusions

Our data clearly show that RNAi machinery takes part in regulating neuron-specific developmental programs, in which the key RNAi components Dicer and Ago-2 display a dynamic chromatin association.

## Methods

### Animals

All experiments were performed in accordance with the European Communities Council Directive (86/609/EEC). Stereotactic injections of retroviruses into the brain of adult mice were approved by the Government of Upper Bavaria. According to German law, the approval of the animal experiments is given by a committee of the Government of Upper Bavaria, which checks (1) the conformity of the proposed study with German animal protection law, and (2) the relevance and ethical conformity. The relevant department in the Government of Upper Bavaria that issued the license for the experiments can be found at:

http://www.regierung.oberbayern.bayern.de/aufgaben/umwelt/verbraucher/ansprech/.

8–12-week-old *Dicer cKO* [27] or C57BL/6 mice were used. Mice were grouped housed in standard cages under a 12 h light/dark cycle and had ad libitum access to food and water.

### Retrovirus preparation

pCAG–GFP–IRES–Cre was generated from the pCAG–GFP vector [66] by replacing the GFP coding sequence with cDNA for GFP and Cre recombinase as well as an internal ribosomal entry site (IRES). Retroviruses were generated as described previously [67]. Virus-containing supernatant was harvested four times every 48 h after transfection and concentrated by two rounds of ultracentrifugation. The obtained viral titers ranged between 10^5^ and 10^8^ colony forming units (cfu)/ml, dependent on the harvest number and the construct.

### Stereotactic injections

All experiments were conducted under the condition that animals have ad libitum access to a running wheel until day 6 post injection. This measure was necessary to enhance proliferation and thus the number of transduced cells. Mice were deeply anesthetized with a mixture of fentanyl (0.05 mg/kg bodyweight), midazolam (5 mg/kg bodyweight) and medetomidine (0.5 mg/kg bodyweight). Anaesthesia was reverted with a mixture of buprenorphine (0.1 mg/kg bodyweight), atipamezole (2.5 mg/kg bodyweight) and flumazenil (0.5 mg/kg bodyweight). Mice were stereotactically injected with 1 µl of the CAG–GFP–IRES–Cre retroviruses (titer 1 × 10^8^ cfu/ml) into the left and right dentate gyrus (coordinates from bregma were −1.9 mm anterior/posterior, ±1.6 mm medial/lateral, −1.9 mm dorsal/ventral from dura). Group size was *n* = 4 mice for each experimental group.

### Tissue processing

Mice were sacrified using CO_2_. They were perfused with PBS, pH 7.4, for 5 min, followed by 4 % paraformaldehyde (PFA) for 5 min. Brains were removed and post fixed in 4 % PFA for 12 h at 4 °C and were subsequently transferred to a 30 % sucrose solution. Forty-micrometer-thick coronal brain sections were cut using a sliding microtome (Leica Microsystems). Brain sections were stored at −20 °C in cryoprotectant solution (0.05 M phosphate butter, 25 % ethylenglycol v/v, 25 % glycerol v/v).

### Histology and counting procedures

Sections were blocked in Tris buffered saline (TBS) supplemented with 3 % normal donkey serum and 0.25 % Triton X-100 for 60 min. Brain sections were incubated in blocking solution containing the primary antibodies at the appropriate dilutions at 4 °C for 48 h. Primary antibodies against the following antigens were used: Dcx (rabbit, 1:250; Abcam ab18723), GFP (chicken, 1:500; Aves Labs GFP-1020), Sox11 (goat, 1:500; Santa Cruz sc-17347). After three washes in TBS, samples were incubated in blocking solution containing the secondary antibody coupled to Cy3, Cy5 or FITC, for 2 h at room temperature. Secondary antibodies were obtained from The Jackson Laboratory and were used at a dilution of 1:250 after resuspension in 200 µl of H_2_O and 200 µl of glycerol. Samples were washed three times with TBS, incubated in 10 mg/ml DAPI (4′,6-diamidino-2-phenylindole; 1:10,000; Sigma-Aldrich) for 10 min, and mounted in Aqua PolyMount (Polysciences). To characterize the phenotype of the retrovirally transduced cells, equidistant sections containing the dentate gyrus were selected and stained. Transduced cells were identified based on the expression of GFP and were analyzed by confocal microscopy for immunoreactivity of the respective markers (*n* > 100 cells per group of at least four animals). The counting procedure was carried out blindly. Confocal single-plane images and *Z*-stacks were taken on a Leica SP5 confocal microscope.

### Cell culture

Neural stem/progenitor cells were isolated from adult (8–12 weeks) hippocampal tissue or subventricular area using the protocol described in [68]. Cells were cultured on poly-d-lysine (PDL; 10 µg/ml; Sigma) and laminin (5 µg/ml; Invitrogen) coated plates in DMEM/F-12 medium (Invitrogen) containing B27 supplement (Invitrogen), 8 mM HEPES buffer, 1× penicillin/streptomycin/fungizone (Invitrogen), 20 ng/ml epidermal growth factor (EGF; PeproTech), and 20 ng/ml basic fibroblast growth factor (FGF2; PeproTech). Cultures were supplemented with growth factors every other day. Differentiation was induced by growth factor withdrawal.

### Dicer deletion by HTN-Cre

For deletion experiments, *Dicer cKO* neural stem/progenitor cells were seeded on PDL/laminin-coated 6-well plates and after 6 h transduced with 1 µM HTN-Cre protein [28]. At 18 h after transduction, growth medium was replaced and the cells expanded to reach the required amount. The Cre-mediated deletion of the Dicer locus was verified by DNA PCR using the following primer pairs: for *Dicer*-*flox*, forward 5′-CCATTTGCTGGAGTGACTCTG-3′ and reverse 5′-TAAATCTGGCAAGCGAGACG-3′ (product size about 400 bp); for *Dicer*-Δ: forward 5′-AGTAATGTGAGCAATAGTCCCAG-3′ and reverse as Dicer-flox (it gives a product only in case of recombination, size is about 350 bp). PCR amplifications were carried out in presence of 5 % (v/v) dimethyl sulfoxide. Differentiation was induced by growth factor withdrawal and harvested for analysis 4 days later. For western blot 20 µg of protein extract were analyzed. Proteins of interest were detected with the following antibodies: rabbit anti-Dicer [1:1000, kindly provided by Chrysi Kanellopoulou [26]; rabbit anti-Dcx (Abcam ab18723); rabbit anti-Actin (1:4000, Abcam ab8227)].

### miRNA-128 overexpression

Neural stem/progenitor cells were isolated from the subventricular zone of 8-weeks old C57BL/6 mice as previously described [69] with the only difference that FGF2 and EGF (Invitrogen) were added both at 20 ng/ml to the B27-supplemented culture medium. Cells were seeded on Matrigel-coated 24-well plates (BD Bioscience) at the density of 1.5 × 10e5 cells per well and transfected 24 h later with a miRNA-128-RFP plasmid or a control miRNA-Ctr-RFP plasmid using Lipofectamine 2000 (Life Technologies; ratio DNA/lipofectamine 1:4). Differentiation was induced by growth factor withdrawal 24 h after transfection. Cells were harvested for analysis 7 days after transfection. Cultures were fixed with 4 % PFA for 15 min. Primary antibodies against the following antigens were used: DCX (guinea pig, 1:2000, Millipore AB5910), GFAP (mouse, 1:500; Sigma G3893). Primary antibodies were applied overnight at 4 °C in 10 % serum, 0.5 % Triton X-100 in PBS. Fluorescent secondary antibody was used according to the manufacturer’s protocol (Jackson ImmunoResearch). Single-plane images were taken on a Zeiss Axioplan fluorescence microscope. The percentage of marker-positive cells among the total RFP-positive population was determined for three wells within three different biological replicates. Approximately from ten to twelve randomly selected fields were evaluated for each condition in each experiment (*n* > 100 cells per experimental group). The counting procedure was carried out blindly.

Neuro2A cells were grown in Opti-MEM medium (Life Technologies). Cells were seeded on 6-well plates and after 24 h transfected with or without miRNA-128-RFP plasmid using Lipofectamine 2000 (Life Technologies; ratio DNA/lipofectamine 1:4). Cells were harvested for analysis 48 h after transfection. For western blot 20 µg of protein extract were analyzed. Proteins of interest were detected with the following antibodies: rabbit anti-Dcx (Abcam ab18723); mouse anti-Gapdh (1:000, clone 6C5, Santa Cruz 32233).

### Dcx knock-down

Neuro2A cells were grown in Opti-MEM medium (Life Technologies). Cells were seeded on 10 cm plates and after 24 h transfected with a shRNA-Dcx plasmid or a control shRNA-Ctr plasmid using Lipofectamine 2000 (Life Technologies; ratio DNA/lipofectamine 1:5). Cells were harvested for analysis 72 h after transfection. shRNA-expressing vectors were previously described [70]. For western blot 20 µg of protein extract were analyzed. Proteins of interest were detected with the following antibodies: rabbit anti-Dcx (Abcam ab18723); rabbit anti-Actin (1:4000, Abcam ab8227).

### RNA isolation and RT-qPCR

Total RNA was isolated using Trizol reagent (Invitrogen). cDNA was synthesized using QuantiTect Reverse Transcription Kit (Qiagen) including DNA elimination step. Real-time qPCRs were performed on a StepOne device (Applied Biosystems). Power SYBR Green PCR Master Mix (Applied Biosystems) was used for detection. Primers for qPCR were as follows: *Sox11* forward, 5′-CCC TGT CGC TGG TGG ATA AG-3′ and reverse, 5′-GGT CGG AGA AGT TCG CCT C-3′; *DCX* forward, 5′-TGC TCA AGC CAG AGA GAA CA-3′ and reverse, 5′-CTG CTT TCC ATC AAG GGT GT-3′; *Srp14* forward, 5′-CAG CGT GTT CAT CAC CCT CAA-3′ and reverse, 5′-GGC TCT CAA CAG ACA CTT GTT TT-3′; *Actin* forward, 5′-TTG CTG ACA GGA TGC AGAAG-3′ and reverse, 5′-ACA TCT GCT GGA AGG TGG AC-3′; *Lin41* forward, 5′-CCC TTC TCC ATT CTC TCG GTG-3′ and reverse, 5′-AGA TGG GGA CAG AGC AGG TGT-3′; *Dicer *forward, 5′-AAT TGG CTT CCT CCT GGT TAT-3′ and reverse, 5′-GTC AGG TCC TCC TCC TCC TC-3′; *Sox2* forward, 5′-GCG GAG TGG AAA CTT TTG TCC-3′ and reverse, 5′-CGG GAA GCG TGT ACT TAT CCT T-3′; *Nestin* forward, 5′-CCT TTC TTC TGT GGC TCA CC-3′ and reverse, 5′-TCA TCA TTG CTG CTC CTC TG-3′. Quantification was normalized to the housekeeping gene *Srp14*.

miRNA cDNA synthesis and amplification was performed with the TaqMan miRNA assay (Life Technologies) allowing the specific detection of the mature miRNA of interest. Real-time qPCRs were performed on the same StepOne device. Quantification in this case was normalized to the small non-coding genes *snoRNA55* or *snoRNA135*. The IDs of the TaqMan Probe used were: miR-125 (000449); let-7c (000379); miR-134 (001186); miR-128 (002216); snoRNA55 (001228); snoRNA135 (001230). Relative expression levels were calculated using the following equation: A = 2[Ct(ref) − Ct(ref-control)] − [Ct(sample) − Ct(sample-control)].

### miRNA overexpressing plasmids

The generation of CAG-RFP-miRNA-IRES-RFP vectors was done as previously described [70]. Additionally the following miRNA128/cloning primers were used:

miRNA128 Fwd: 5′-TGCTG tca cag tga acc ggt ctc ttt GTT TTG GCC ACT GAC TGA CAA AGA GAC CTT CAC TGTGA-3′.

miRNA128 Rew: 5′-CCT GTCA CAG TGA AGG TCT CTT TGT CAG TCA GTG GCC AAAAC aaa gag acc ggt tca ctg tgaC-3′ (in lower case is the mature miRNA128 sequence).

### Chromatin binding assay

The procedure was used essentially as previously described [23, 39]. Neural stem/progenitor cells (7× confluent 10 cm plates of undifferentiated cells or 20× 10 cm plates of 3-days differentiating cells) were washed with cold PBS, detached from the plates with Accutase (Millipore), washed again with PBS and finally resuspended in 6 ml of cold PBS. One tenth of the cell suspension (control fraction, C), was centrifuged at 400*g* and resuspended in RIPA buffer [150 mM Tris–HCl, pH 8.0, 150 mM NaCl, 0.5 % DOC, 0.1 % (w/v) SDS, 1 % (v/v) NP-40, Roche protease inhibitor cocktail] and left 30 min on ice. The remaining fraction was lysed for 15 min on ice in cold CSKI buffer [10 mM Pipes, pH 6.8, 100 mM NaCl, 1 mM EDTA, 300 mM sucrose, 1 mM MgCl_2_, 1 mM DTT, 0.5 % (v/v) Triton X-100, Roche protease inhibitor cocktail, 1 mM PMSF]. The cell lysate was divided into two portions, which were centrifuged at 500*g* at 4 °C for 3 min. The supernatants (S1 fraction), which contain Triton-soluble proteins, were further analyzed. One of the pellets was washed twice in CSKI buffer and then resuspended in RIPA buffer (the P1 fraction). The second pellet, after 2× CSKI washes, was resuspended in CSK II buffer (10 mM Pipes pH 6.8, 50 mM NaCl, 300 mM sucrose, 6 mM MgCl_2_, 1 mM DTT, Roche protease inhibitor cocktail), treated with DNase for 30 min followed by extraction with 250 mM NH_2_SO_4_ for 10 min at 25 °C. The sample treated with DNase (Promega) and salt was then centrifuged at 1200*g* for 6 min at 4 °C and the supernatant (S2 fraction), and pellet (P2 fraction), were collected. P2 after 2× washes in CSKII buffer was also resuspended in RIPA buffer. 20 µg of all fractions were analyzed by immunoblotting. Proteins of interest were detected with the following antibodies: mouse anti-RNA Polymerase II (1:1000, clone 4H8, Abcam 5408); rabbit anti-Dicer [1:1000, kindly provided by Chrysi Kanellopoulou [26]; rat anti-Argonaute-2 (1:50; clone 6F4), kindly provided by Elisabeth Kremmer [71]; mouse anti-β-catenin (1:2000, BD Biosciences 610153); mouse anti-Gapdh (1:1000, clone 6C5, Santa Cruz 32233)].

## Additional files

**Additional file 1: Figure S1.** *Dicer* deletion in adult neural stem cells. a–b The samples analyzed were adult neural stem cells *Dicer flox/flox (f/f)* or *Dicer −/−*. Dicer deletion was obtained by transducing the *Dicer f/f* cells with HTN-Cre protein. a) Representative *picture* of PCR genotyping of adult neural stem cells *Dicer f/f* and *Dicer −/−*. Efficient *Dicer* deletion was obtained by transducing the *Dicer f/f* cells with HTN-Cre protein. Primers-Δ only amplify in case of recombination. b) Quantitative RT-PCR. The cycle threshold numbers are plotted for the indicated genes. *Srp14* is a housekeeping gene. The cycle threshold numbers are reciprocally correlated to the amount of template at the start of linear amplification. *Nestin*, *Sox2* and *Srp14* expression levels are very similar in both *Dicer f/f* or *Dicer −/−* cells. The mean values from 3 independent experiments are shown. Error bars indicate the standard error of the mean.
**Additional file 2: Figure S2.** miRNA-128 levels in adult neural progenitors cells. Shown is a Taqman quantitative RT-PCR on undifferentiated (undiff) or differentiating (diff; 4 days after growth factor withdrawal) adult neural progenitor cells. Levels of mature miR-128 were calculated as fold change relative to the housekeeping gene *snoRNA55*. n = 3, bars represent the mean ± standard error of the mean. Three independent biological samples have been analyzed.
**Additional file 3: Figure S3.** miR128 over-expression reduces Dcx protein in N2 cells. Western blot showing the Dcx (arrow) and Actin (loading control) protein levels in (from far right to left): mock (control transfection without miRNA-128-RFP expressing plasmid), miRNA-128 (cells treated with miRNA-128-RFP expressing plasmid), shRNA–Dcx (cells treated with shRNA targeting Dcx) and shRNA-Ctr (cells treated with control shRNA). The band corresponding to Dcx is reduced in both shRNA–Dcx and miR-128 samples. Dcx KD = Dcx knockdown; miR-128 OE = mirR-128 over-expression; MW = Molecular weight marker.

## Abbreviations

SEM: standard error of the mean
ChIP-seq: Chromatin immunoprecipitation followed by next generation sequencing
NSCs: neural stem cells
DG: dentate gyrus
SVZ: subventricular zone
Dcx: Doublecortin
RNAi: RNA interference
miRNAs: microRNAs
siRNAs: small interfering RNAs
piRNAs: Piwi-interacting RNAs
dpi: days post injection
DAPI: 4′,6-diamidino-2-phenylindole
PDL: poly-d-lysine
FGF: fibroblast growth factor
