# Supplementary figures and images for: RNA interference machinery-mediated gene regulation in mouse adult neural stem cells
Source: BMC Neurosci. 2015 Sep 19;16:60. doi: 10.1186/s12868-015-0198-7 (PMC4575781; doi:10.1186/s12868-015-0198-7)

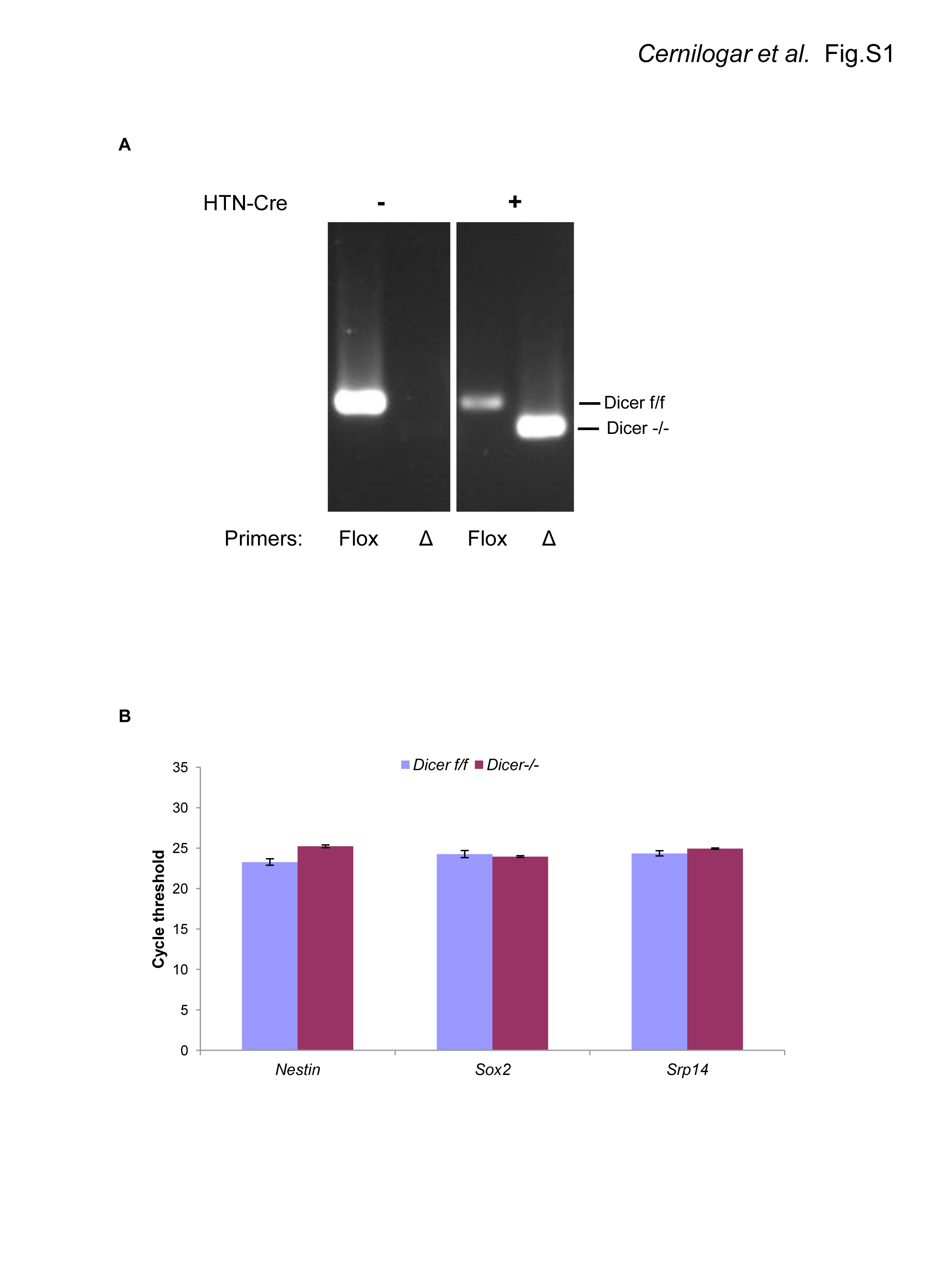

Supplement: Supplementary file 1 — Additional file 1: Figure S1. Dicer deletion in adult neural stem cells. a–b The samples analyzed were adult neural stem cells Dicer flox/flox (f/f) or Dicer −/−. Dicer deletion was obtained by transducing the Dicer f/f cells with HTN-Cre protein. a) Representative picture of PCR genotyping of adult neural stem cells Dicer f/f and Dicer −/−. Efficient Dicer deletion was obtained by transducing the Dicer f/f cells with HTN-Cre protein. Primers-Δ only amplify in case of recombination. b) Quantitative RT-PCR. The cycle threshold numbers are plotted for the indicated genes. Srp14 is a housekeeping gene. The cycle threshold numbers are reciprocally correlated to the amount of template at the start of linear amplification. Nestin, Sox2 and Srp14 expression levels are very similar in both Dicer f/f or Dicer −/− cells. The mean values from 3 independent experiments are shown. Error bars indicate the standard error of the mean. [file 12868_2015_198_MOESM1_ESM.tiff]

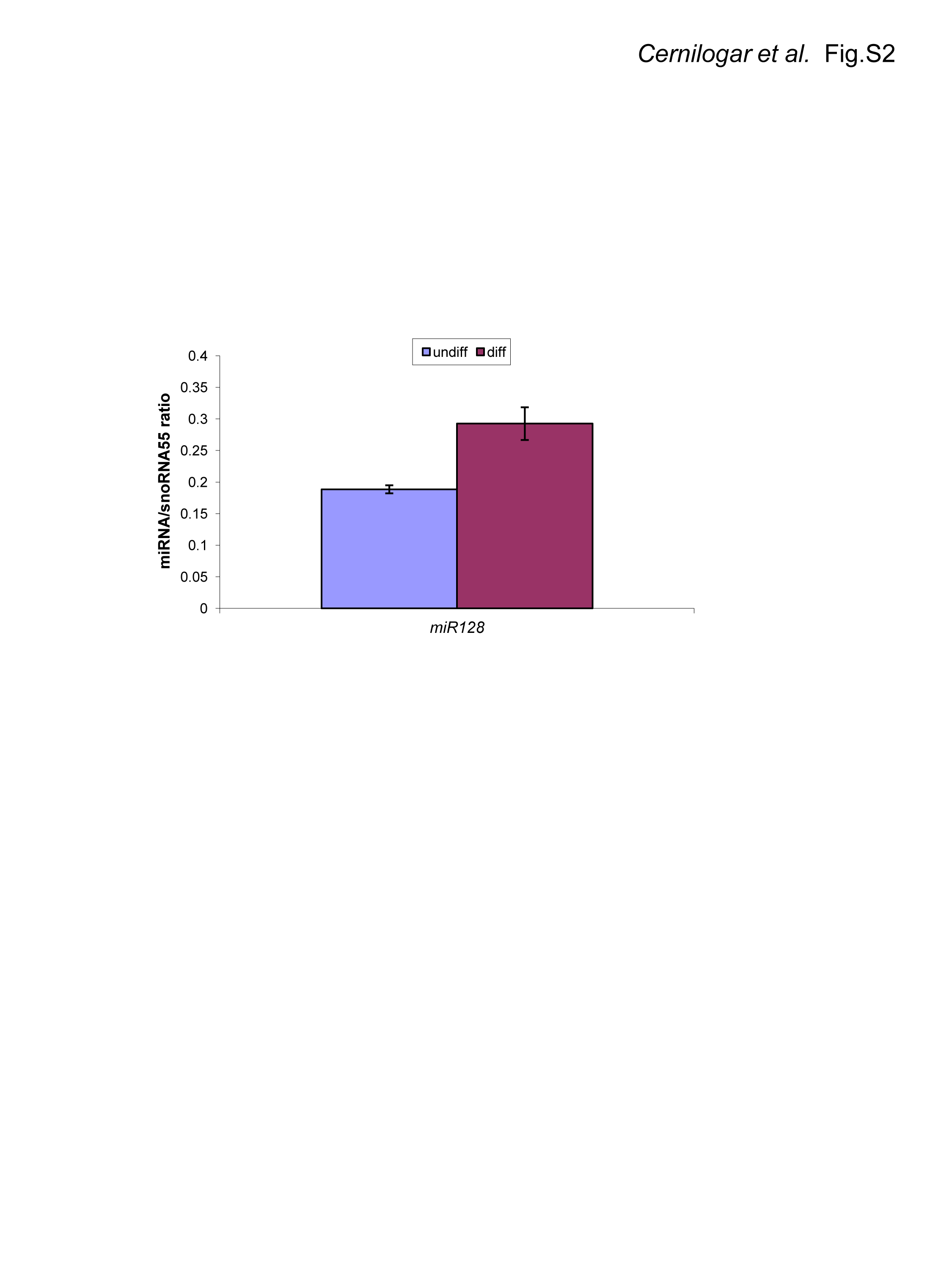

Supplement: Supplementary file 2 — Additional file 2: Figure S2. miRNA-128 levels in adult neural progenitors cells. Shown is a Taqman quantitative RT-PCR on undifferentiated (undiff) or differentiating (diff; 4 days after growth factor withdrawal) adult neural progenitor cells. Levels of mature miR-128 were calculated as fold change relative to the housekeeping gene snoRNA55. n = 3, bars represent the mean ± standard error of the mean. Three independent biological samples have been analyzed. [file 12868_2015_198_MOESM2_ESM.tiff]

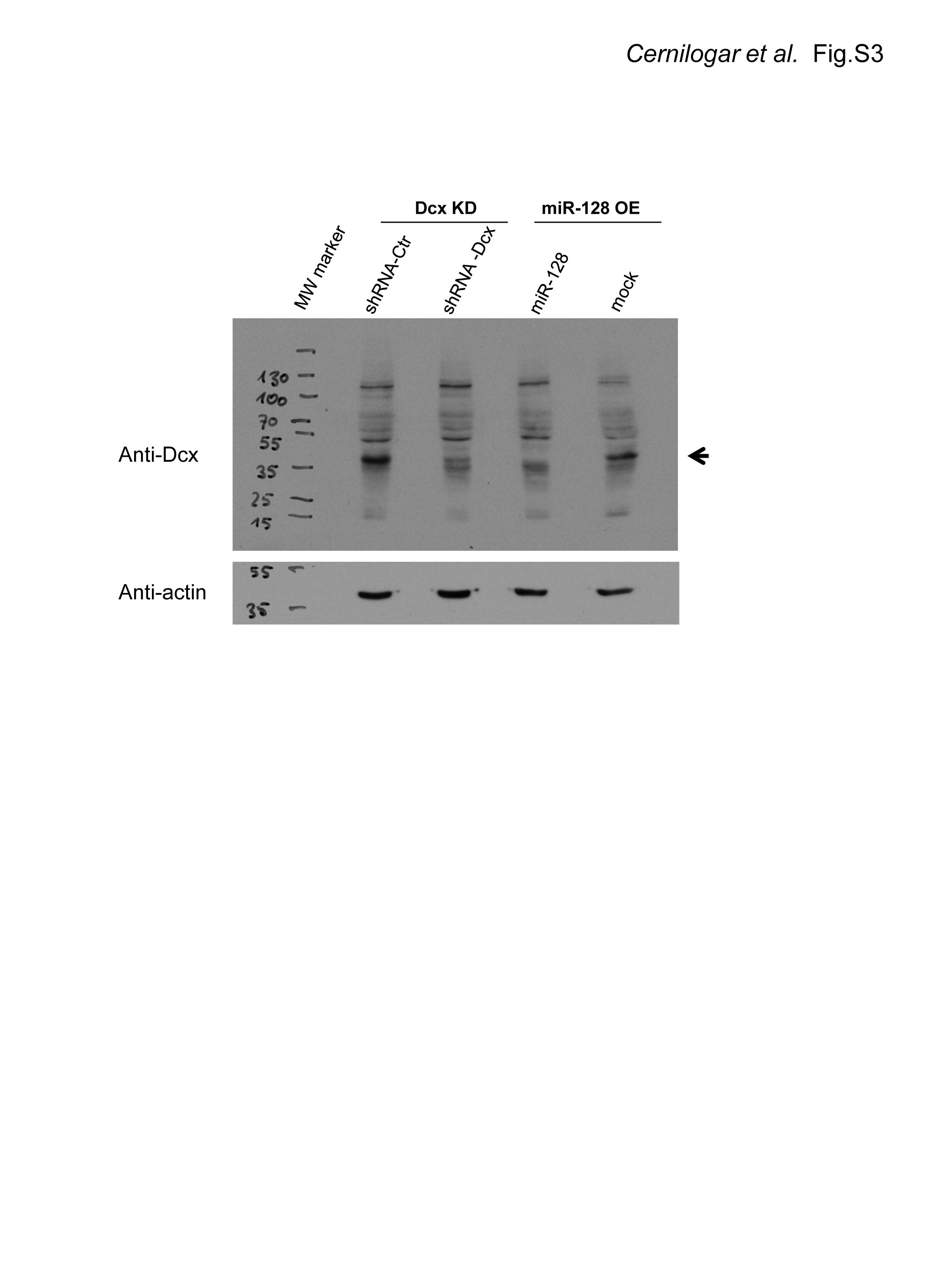

Supplement: Supplementary file 3 — Additional file 3: Figure S3. miR128 over-expression reduces Dcx protein in N2 cells. Western blot showing the Dcx (arrow) and Actin (loading control) protein levels in (from far right to left): mock (control transfection without miRNA-128-RFP expressing plasmid), miRNA-128 (cells treated with miRNA-128-RFP expressing plasmid), shRNA–Dcx (cells treated with shRNA targeting Dcx) and shRNA-Ctr (cells treated with control shRNA). The band corresponding to Dcx is reduced in both shRNA–Dcx and miR-128 samples. Dcx KD = Dcx knockdown; miR-128 OE = mirR-128 over-expression; MW = Molecular weight marker. [file 12868_2015_198_MOESM3_ESM.tiff]
